# 2D Coordination Network of Trioxotriangulene with Multiple Redox Abilities and Its Rechargeable Battery Performance

**DOI:** 10.3390/ijms21134723

**Published:** 2020-07-02

**Authors:** Tsuyoshi Murata, Taro Koide, Hirofumi Nobukuni, Ryotaro Tsuji, Yasushi Morita

**Affiliations:** 1 Department of Applied Chemistry, Faculty of Engineering, Aichi Institute of Technology, Yachigusa 1247, Yakusa, Toyota, Aichi 470-0392, Japan; koide@mail.cstm.kyushu-u.ac.jp (T.K.); nobukuni@aitech.ac.jp (H.N.); 2 Department of Chemistry and Biochemistry, Graduate School of Engineering, Kyushu University, Moto-oka 744, Nishi-ku, Fukuoka-shi, Fukuoka 819-0395, Japan; 3 Material Solutions New Research Engine, KANEKA Corporation, Suita, Osaka 565-0871, Japan; Ryotaro.Tsuji@kaneka.co.jp

**Keywords:** metal–organic framework, organic rechargeable battery, multistage redox ability, tridentate ligand, polycyclic aromatic molecule

## Abstract

A three-fold symmetric trioxotriangulene derivative with three pyridyl groups as coordinating sites was designed and synthesized. In a cyclic voltammetry measurement, the trioxotriangulene skeleton exhibited a multi-stage redox ability from neutral radical to radical tetra-anion species. In the zinc complex of monoanion species, three pyridyl groups coordinated to the zinc ion to build up a two-dimensional coordination network with a cavity larger than 12 Å in diameter. This complex was utilized as a cathode active material of a lithium ion battery, and it exhibited a capacity of ca. 60 mAh g^−1^ per the weight of the active material with a stable cycling performance up to 1000 cycles. This work shows that the coordination network formed by the trioxotriangulene-based ligand was effective in the improvement of cycle performance of the organic rechargeable battery.

## 1. Introduction

With an increasing demand for high performance energy storage systems, organic rechargeable batteries (ORB), in which a redox active organic molecule is utilized as an electrode-active material instead of inorganic metal oxides, has being attracting much attention [1,2,3,4,5,6,7,8,9,10,11,12,13,14,15]. Organic electrode-active materials are composed of ubiquitous elements, and have lower environmental effects as compared to inorganic metal oxide (Co, Ni, Mn and etc.) electrodes in conventional lithium-ion batteries (LIBs). In addition, an intrinsic absence of a large amount of O^2−^ ion in organic electrode-active materials plays a vital role in an increase in safety of the battery by preventing O_2_ generation from over-charge, which is in significant contrast to the cases of conventional LIBs using inorganic metal oxide as electrode-active materials. Organic electrode-active materials can be engineered at the molecular level, and recent investigations have improved electrochemical performances of ORBs. On the other hand, some problems specific to organic materials remain. One of the most significant issues is the decrease in capacity during the charge–discharge cycle, mainly due to the decomposition of organic materials and their dissolution into electrolyte solution [4,16,17,18].

The metal–organic framework (MOF) is a class of porous crystalline materials constructed by the coordination network of metal-ions and organic linkers, and possesses a wide potential in various applications [19,20,21,22,23,24,25,26]. When an electrode-active molecular moiety is incorporated into the MOF as a linker, the cycle performance of its ORB is expected to be improved, since the dissolution is likely to be suppressed by the rigid and flexible coordination bonds. Furthermore, a cavity in the coordination network can be utilized as a space for Li-ion to increase the efficiency and speed of giving and receiving of electrons in charge–discharge processes. From these viewpoints, redox-active MOFs were widely investigated as cathode and anode active materials of LIB [27,28,29,30,31,32,33,34,35,36,37,38].

**Scheme 1:**
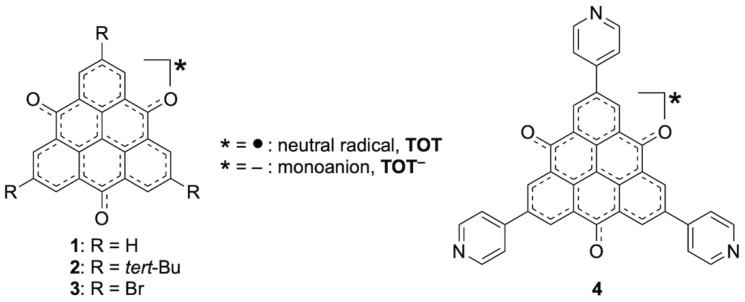
Molecular structures of trioxotriangulene (**TOT**) and pyridine-incorporated **TOT 4**.

Trioxotriangulene (**TOT 1**, Scheme 1) is a fused-polycyclic hydrocarbon [39,40] functionalized with three oxo-groups, and its neutral radical is highly stable even without steric protections due to the delocalization of electronic-spin [41]. Our recent investigation revealed various intriguing physical properties and functions of **TOT** derivatives based on the redox activity and self-assembling ability by π-stacking [41,42,43,44,45,46,47,48]. We also demonstrated that **TOT** exhibits a four-stage redox ability from neutral radical to radical tetraanion species. The generation of polyanionic species realized a high capacity ORB using the *tert*-butyl (*tert*-Bu) and bromo derivatives (**2** and **3**, respectively) as cathode-active materials [39,49,50]. The ORB of **2** showed a high initial capacity per the weight of active material (*C*_TOT_) of 311 mA h g^−1^; however, the capacity decreased with repeating charge–discharge processes (*C*_TOT_ = 73 mA h g^−1^ at 100 cycles) due to elution of the active material into the electrolyte solution. On the other hand, in the case of **3**, in which molecules were connected by halogen bonds in the crystal, elution of the active material into the electrolyte was sufficiently suppressed, and cycle performance was greatly improved (*C*_TOT_ = 225 mA h g^−1^ at 1st cycle and *C*_TOT_ = 159 mA h g^−1^ at 100th cycle). These results suggest that in addition to the chemical stability of the redox species, suppression of the solubility of active materials by intermolecular interactions is effective for improving cycle characteristics [51,52,53,54,55]. In this paper, we have designed and synthesized a novel **TOT** derivative **4** with three 4-pyridyl groups introduced at the 2-,6- and10-positions of the **TOT** skeleton, preserving the three-fold symmetry as the first ligand based on the **TOT**s for metal complexes (Scheme 1). The zinc(II) complex of **4^−^** monoanion constructed a 2D coordination network with a cavity size of ~12 Å. We also investigated the charge–discharge behavior of an ORB using the **4^−^**–Zn(II) complex as a cathode-active material.

## 2. Results

### 2.1. Synthesis and Structures of TOT with Three Pyridyl Moieties

**Scheme 2:**
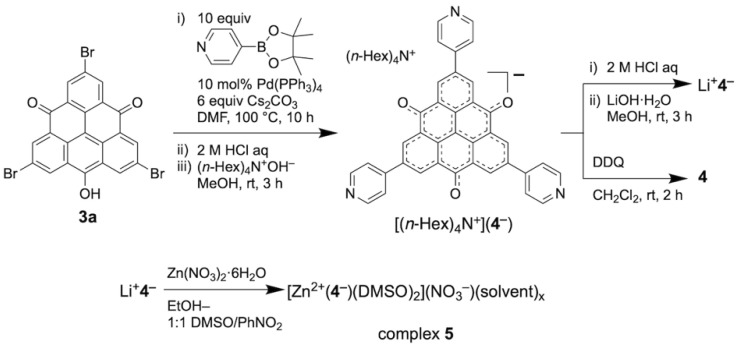
Synthetic procedure of **4^−^** salts, neutral radical **4** and zinc(II) complex **5**.

Three pyridyl groups were introduced into **TOT** by the Suzuki–Miyaura cross-coupling reaction of tribromo **TOT** derivative **3a** [56] with 4-(4′,4′,5′,5′-tetramethyl-1′,3′,2′-dioxaborolan-2′-yl)pyridine under the presence of a Pd catalyst (Scheme 2). The cross-coupling product of **4^−^** was isolated as a salt with tetra-*n*-hexylammonium, [(*n*-Hex)_4_N^+^](**4^−^**). The **4** neutral radical was obtained as an insoluble black solid by chemical oxidation of [(*n*-Hex)_4_N^+^](**4^−^**) in CH_2_Cl_2_ with 2,3-dichloro-5,6-dicyano-*p*-benzoquinone (DDQ). This solid was stable at room temperature under air for a long period of time. ^1^H NMR spectrum of the monoanion **4^−^** salt in dimethyl sulfoxide (DMSO)-*d*_6_ shows a simple spectrum with three signals at 8.90, 8.67 and 7.82 ppm (Figure S1), which are assignable as the C–H protons at the α-positions in the **TOT** skeleton and at 2- and 3-positions in the pyridyl groups, respectively.

**Figure 1:**
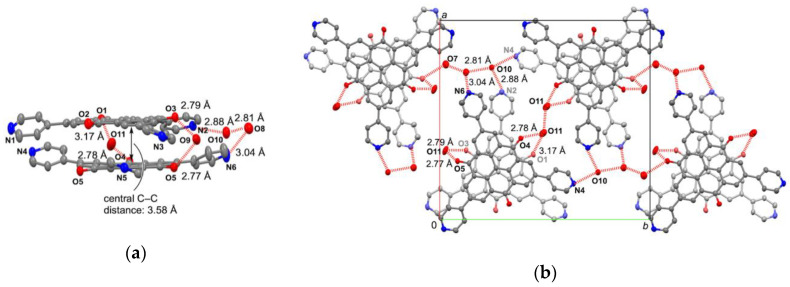
Crystal structure of the weak π-dimeric structure of [(*n*-Hex)_4_N^+^](**4^−^**). (**a**) Side view; (**b**) hydrogen-bonded network of **4^−^** and water molecules, and overlap pattern. Color code: carbon (gray), nitrogen (blue), oxygen (red).

In the crystal structure of [(*n*-Hex)_4_N^+^](**4^−^**) salt, the asymmetric unit includes crystallographically two kinds of **4^−^** molecules. The crystal also included crystallographically five independent water molecules and one dimethoxyethane (DME) molecules (Figure S3). The component ratio in this crystal was [(*n*-Hex)_4_N^+^](**4^−^**):H_2_O:DME = 2:5:1. The **TOT** skeletons of both **4^−^** molecules possessed nearly planar structures with C–C and C–O bond lengths of 1.36–1.48 Å and 1.23–1.26 Å (Table S1), respectively, which are comparable to those of pristine **1^−^** [41], and the whole molecular structures of **4^−^** were close to a three-fold symmetry. This result suggests that the minus charge on **4^−^** widely delocalizes around the highly symmetric **TOT** skeleton. The pyridyl groups were twisted to the **TOT** skeleton by 23.3–32.6°. As shown in Figure 1a, the **4^−^** molecule was stacked to form a weak π-dimeric structure with the aid of hydrogen bonds via crystalline water molecules. The π-dimer had a twisting stacking pattern (Figure 1b), which are also seen in several **TOT** neutral radical crystals [41,42], and the distance between central carbon atoms within the π-dimer was 3.58 Å. Hydrogen bonds via water molecules were also observed between the π-dimers, forming a 2D network (Figure 1b).

### 2.2. Electrochemical Properties of TOT with Three Pyridyl Moieties

**Figure 2:**
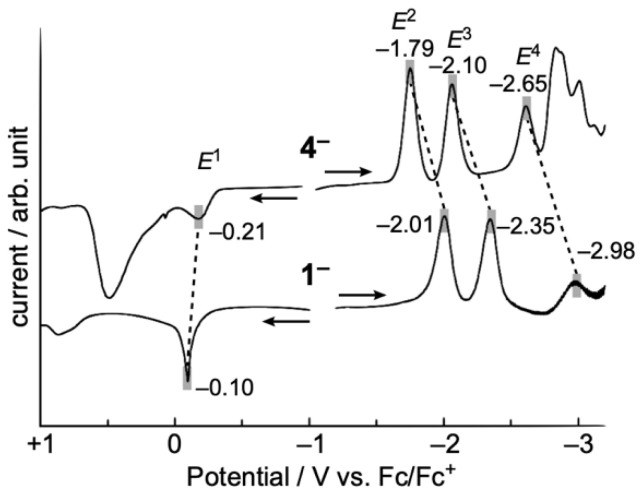
Differential pulse voltammetry (DPV) of **1** and **4** in DMF solution. The results were calibrated with the ferrocene/ferrocenium couple (Fc/Fc^+^). Due to poor solubility, corresponding monoanion salts, [(*n*-Bu)_4_N^+^](**1^−^**) and [(*n*-Hex)_4_N^+^](**4^−^**), respectively, were used for the measurements. The waves observed at the higher potential than *E*^1^ and lower potential than *E*^4^ would be derived from the aggregates or decomposition of the corresponding products, because they were irreversible by cyclic voltammetry (Figure S2).

**Figure 3:**
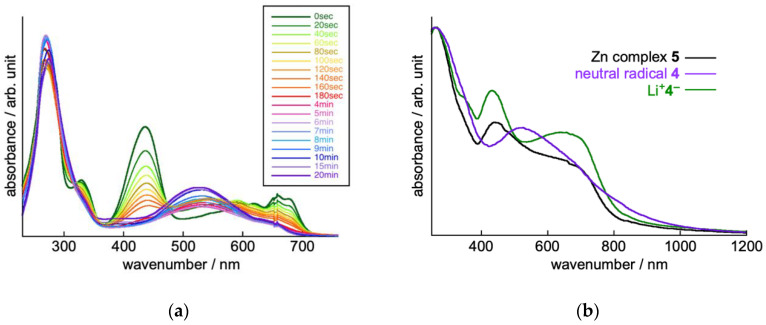
(**a**) Changes in the UV-Vis/near-IR spectra during the electrochemical oxidation of [(*n*-Hex)_4_N^+^](**4^−^**) in CH_2_Cl_2_ containing 0.1 M (*n*-Bu)_4_N^+^PF_4_. The green and purple lines show the spectra recorded before and after the application of an external potential of +1.2 V vs. Ag/AgNO_3_; (**b**) Solid-state electronic spectra of Li^+^**4^−^**, neutral radical **4** and zinc(II) complex **5** in potassium bromide (KBr) pellets. All spectra were measured at room temperature.

The electronic effect of pyridyl groups on the redox ability of **TOT** was evaluated by differential pulse voltammetry (DPV) of [(*n*-Hex)_4_N^+^)](**4^−^**) in *N*,*N*-dimethylformamide (DMF) (Figure 2, see also Figure S2). The **4^−^** salt exhibited one oxidation (*E*^1^) and three reduction (*E*^2–4^) waves. The potentials of *E*^2^–*E*^4^ of **4^−^** showed positive shifts by 0.2–0.3 V from those of pristine **1** due to the electron-deficient feature of the pyridine group. Figure 3a shows the temporal change of the UV-Vis/near-infrared (IR) absorption spectrum of [(*n*-Hex)_4_N^+^)](**4^−^**) under the electrochemical oxidative condition at +1.2 V (vs. Ag/Ag^+^) in CH_2_Cl_2_. At the initial state, the monoanion **4^−^** shows absorption bands of *λ*_max_ = 440 and 680 nm^−1^, the latter of which corresponds to the highest occupied molecular orbital–lowest unoccupied molecular orbital (HOMO–LUMO) transition. As the electrochemical oxidation proceeded, these absorption bands gradually disappeared, and a new broad absorption band with *λ*_max_ of ~530 nm appeared. The feature of the oxidized state is in good agreement with the **4** neutral radical synthesized by the chemical oxidation (Figure 3b).

### 2.3. Crystal Structure of Zinc(II) Complex 5

**Figure 4:**
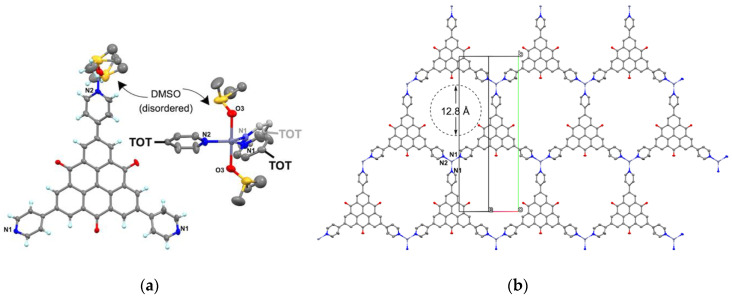
Crystal structure of **5**. (**a**) Molecular structure of **TOT 4^−^** and the coordination sphere around the zinc(II) ion; (**b**) 2D coordination network with a cavity of ca. 12.8 Å in diameter. In (**b**), hydrogen atoms, DMSO and disordered NO_3_^−^, and solvent molecules are omitted for clarity. Color code: carbon (gray), nitrogen (blue), oxygen (red), sulfur (yellow), zinc (blue gray).

**Figure 5:**
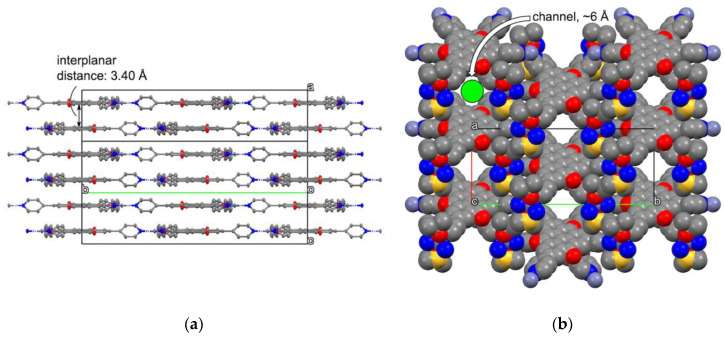
Crystal packing of **5**. (**a**) Side view of the 2D coordination sheet showing the π-stacking motif of **TOT** monoanion; (**b**) Crystal packing viewed along the *c*-axis in the space filling model. Green circle shows the 1D channel filled with NO_3_^−^ and solvent molecules. Hydrogen atoms, disordered NO_3_^−^ and solvent molecules are omitted for clarity. Color code: carbon (gray), nitrogen (blue), oxygen (red), sulfur (yellow), zinc (blue gray).

A zinc(II) complex of the monoanion species **4^−^** was prepared by diffusion of solutions of Li^+^**4^−^** salt in 1:1 DMSO/nitrobenzene and zinc nitrate in EtOH to give a [Zn^2+^(**4^−^**)(DMSO)_2_](NO_3_^−^)(solvent)_x_ complex **5** as green crystals (Scheme 2). Complex **5** was stable under air at room temperature and was insoluble in all organic solvents. In the crystal structure, the **4^−^** molecule possessed a rotation axis, and half of the **TOT** skeleton was crystallographically independent (Figure 4a or Figure S4). The intramolecular C–C and C–O bond lengths were close to those in the [(*n*-Hex)_4_N^+^](**4^−^**) (Table S1). The solid-state electronic spectra of complex **5** is very similar to Li^+^**4^−^**, suggesting that the **TOT** is in the monoanion state (Figure 3b). The zinc(II) center formed a five-coordination sphere with a trigonal bipyramidal geometry composed of three pyridyl groups and two DMSO molecules (Figure 4a). Bond lengths of Zn–N were 2.022(5) and 2.033(5) Å, and that of Zn–O was 2.125(5) Å. The coordination between zinc(II) ion and two pyridyl groups on **4^−^** formed a zigzag chain, and the other pyridyl group coordinated to the zinc(II) ion. Thus, the **4^−^** molecule can be regarded as a three- connected node constructing a 2D coordination sheet with a 6^3^-honeycomb-type topology (Figure 4b). The sheet had a cavity with a diameter of ca. 12.8 Å, and formed a π-stacking motif along the *c*-axis with a face-to-face distance of 3.40 Å (Figure 5a). A small 1D channel of ~ 6 Å was formed parallel to the *c*-axis, and it was filled with NO_3_^−^ and crystalline solvent molecules, the latter two of which were disordered (Figure 5b). In order to investigate the stability of the coordination network, the powder X-ray diffraction (XRD) measurements of the isolated complex **5** was performed (Figure S5). The observed XRD pattern was different from the simulated one from the crystal structure, indicating that the crystal structure of **5** changed by the elimination of solvent molecules from the cavity. On the other hand, this complex was insoluble in any organic solvent even after the elimination of the solvent. This is quite different from the fact that the Li^+^**4^−^** salt dissolves in highly polar solvents such as methanol or DMSO, and implies that the **4^−^** molecules are strongly bound to each other through coordination with the zinc(II) ion even after the crystal structure is deformed.

### 2.4. Solid-State CV and Battery Performance of Zn(II) Complex 5

**Figure 6:**
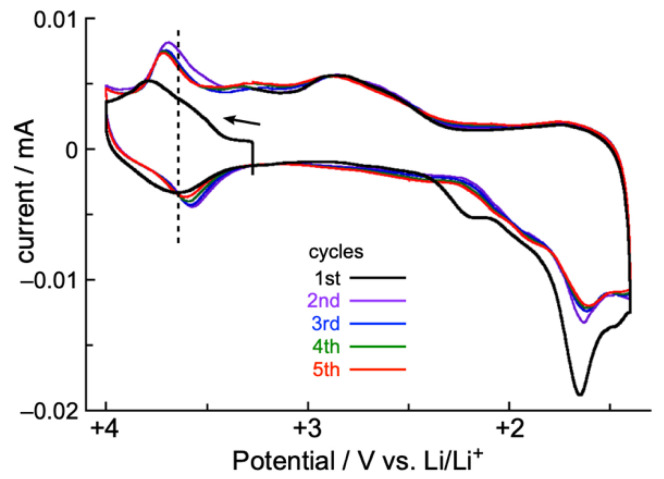
Solid-state cyclic voltammogram (CV) of zinc(II) complex **5** (V vs. Li/Li^+^). Coin-type cell with a cathode composed of 10:10:80 (wt%) of **5**, poly vinylidene difluoride (PVDF) and acetylene black, (AB), respectively; a Li metal anode; and an electrolyte solution of 1 M LiPF_6_ in 3:7 *v*/*v* of ethylene carbonate/diethyl carbonate (EC/DEC). The scan rate was 0.1 mV/s.

Cyclic voltammogram (CV) of complex **5** in the solid state was measured by the coin-type cell (vide infra) and showed several redox waves (Figure 6). The first oxidation wave assignable to the change from monoanion to neutral radical of the **TOT** skeleton was observed as a reversible wave at *E*_1/2_ = 3.65 V vs. Li/Li^+^, which corresponds to −0.04 V vs. Fc/Fc^+^. The first oxidation potential was slightly higher than that observed for [(*n*-Hex)_4_N^+^)](**4^−^**) in the DMF solution (*E*^1^ = −0.21 V vs. Fc/Fc^+^, Figure 2). This result suggests that electrochemical oxidation of **5** affords the neutral radical state of the **TOT** skeleton, even in the zinc(II) complex. The slightly higher potential shift might be derived from the coordination to the zinc(II) ion. At a lower potential, several reduction peaks were observed at 1.5–2.2 V vs. Li/Li^+^, which correspond to −2.2 to −1.5 V vs. Fc/Fc^+^, respectively. These peaks were assignable to changes to the polyanionic species (*E*^2^–*E*^4^ in Figure 2), respectively.

**Figure 7:**
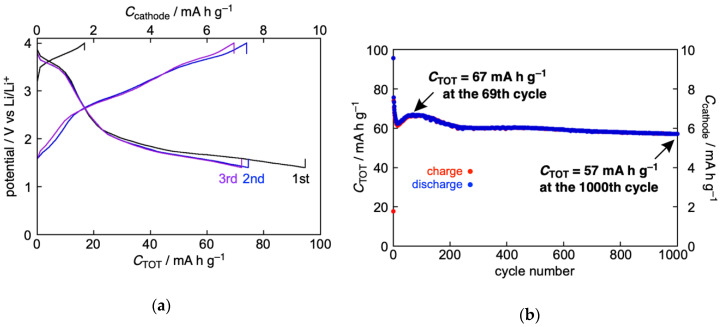
Charge–discharge properties of the coin-type cell of the zinc(II) complex **5**. (**a**) The three-cycle measurement at 1 C between 1.4 and 4.0 V vs. Li/Li^+^; (**b**) The cycle performance of the cell.

The poor solubility of complex **5** due to the strong coordination interaction is considered to be advantageous for the improvement of cycle performance as a cathode-active material of ORBs, even after deformation of the cavity shown in the single crystal structure (Figure 4b or Figure 5b). Here, we prepared a coin-type cell composed of a cathode involving 10 wt% of **5** as an active material with 10 wt% of poly vinylidene difluoride (PVDF) and acetylene black (AB, 80 wt%). The charge–discharge measurement of the cell was performed at 1 C in the voltage range of 1.4 to 4.0 V vs. Li/Li^+^. In the discharge curve, broad plateaus were observed around 3.5 V and 2.0–1.5 V vs. Li/Li^+^ (Figure 7a), and their potentials are close to the redox peaks in the solid-state CV (Figure 6). Thus, these plateaus can be attributed to the electron transfer between neutral radical–monoanion and monoanion–polyanion species of the **TOT** skeleton, respectively. The average discharge voltage was estimated to be 2.16 V. The discharge capacity of the cathode including only 10 wt% of **5** (*C*_cathode_) at the first cycle was of 9.6 mA h g^−1^, from which the capacity of **5** itself (*C*_TOT_) was calculated as 96 mA h g^−1^, assuming that PVDF and AB do not contribute to charge and discharge. The initial *C*_TOT_ value was ~75% of the theoretical value of **5**, 128 mA h g^−1^, which was calculated assuming the four-electron redox of the **TOT** skeleton. The discharge capacity *C*_cathode_ drastically decreased to ~6 mA h g^−1^ (~60 mA h g^−1^ of *C*_TOT_) within initial several cycles, and the capacity was almost maintained up to the 1000th cycle (Figure 7b). It has been observed that the accommodation of electrons in the vicinity of the surface of active materials contributes to the super-capacity [57]. A similar capacity decrease was also observed in the cell of **2** [49], and there is a possibility that the surface of complex **5** or the electrolyte solution would contribute to a part of the capacity in the initial several cycles. Based on the maximum *C*_TOT_ of 67 mA h g^−1^ at the 69th cycle, the capacity retention at the 1000th cycle was 85% (Figure 7b). Thus, the initial capacity of the cell of complex **5** was smaller than **2** and **3**; however, the cycle performance was substantially superior to those of **2** and **3** (43% and 71% at the 100th cycle, respectively) [49,50]. From this result, we speculate that the coordination of **4^−^** to the zinc(II) ion was robust during charge–discharge process, and elution of the active material was well suppressed even in the polyanionic states.

## 3. Discussion

**Figure 8:**
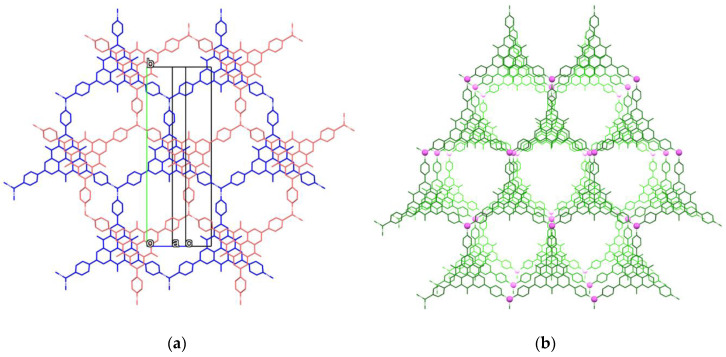
(**a**) Overlap view of the 2D coordination sheet in **5**. The red sheet is on the upper side, and the blue one is on the lower side. (**b**) A model packing structure with a large 1D channel designed by ideally parallel stacking of a 2D coordination sheet in **5**. The green molecular skeletons represent **4** species, and the magenta balls are metal ions. The sheets on the lower side are drawn in lighter colors.

In this work, we would like to discuss the expected effects of the coordination network and possible strategies for further improvement of the battery performance. The X-ray crystal structure analysis of **5** revealed that the coordination bonds established a 2D network with a cavity ~12 Å in size. Compared with the previously reported MOFs used as the active materials [27,28,29,30,31,32,33,34,35,36,37,38] and Stokes radii of the Li ion (~4.8 Å in propylene carbonate) [58], the cavity in the 2D coordination sheet of **5** is large enough to be used as a space for storage and transfer of Li ions. On the other hand, in the π-stacking structure, the sheets stacked non-parallelly, and the coordinating sites in the next sheet partially cover the cavity (Figure 8a) and, thus, the 1D channel narrowed to ~6 Å (Figure 5b). Furthermore, the XRD of complex **5** after the isolation suggests that the crystal structure decayed by the elimination of the guest–solvent molecules (Figure S5). It is also assumed that a similar structural deformation of the MOF would be caused by the intercalation/release of Li ions and NO_3_^−^ during charge–discharge processes. In addition, the elimination of solvent molecules can occur even on the preparation of the cathode through grinding, dispersion in an organic solvent and vacuum-drying. Therefore, the small capacity of the battery (~50% of the theoretical value) is probably due to the collapse of the cavity for the transfer and storage of Li ions. On the other hand, the result that the capacity after the initial decrease was maintained up to 1000 cycles indicates that suppression of dissolution of the active material with the aid of robust coordination of **4^−^** is effective for the improvement of cycle performance, even when the MOF was collapsed.

In order to improve the efficiency of charge–discharge, it might be necessary to stack the 2D coordination sheets in a parallel manner and to maintain a large 1D channel structure (Figure 8b). In complex **5**, DMSO is a coordinate for the upper and lower sites of the trigonal bipyramidal coordination structure (Figure 4a). For example, it is considered to be effective to introduce a linear bidentate ligand (pillar ligand) such as pyrazine as a linker between the 2D coordination sheets (Figure 8b) [59,60,61,62]. The construction of such a 3D coordination network would be effective in preventing the collapse of the MOF and cavity during intercalation/release of guest molecules and ions. In complex **5**, the **TOT** skeleton was a close-shell monoanion state, and did not form a 1D π-stacking column. Therefore, **5** was an insulator, and it was necessary to add 80 wt% of conductive additive (AB) to the cathode, causing a small capacity of the cathode (*C*_cathode_). This has been one of the most difficult issues in ORBs using other redox-active molecules [28,32,34,37,63,64]. In the pillared-layer structure in Figure 8b, the **TOT** skeleton can form a 1D π-stacking structure. As revealed in our previous works, the π-stacking column of the **TOT** neutral radical provides an effective electron transfer pathway [42,43,48] and, thus, the electrical conductivity can be imparted to the active material itself. The study to solve these problems is currently underway in our laboratory.

## 4. Materials and Methods

### 4.1. Synthesis and Characterization

A detailed synthetic method for the tribromo-**TOT** derivative **3a** is described in our previous work [56]. DMF, used for the synthesis and solution state DPV/CV measurements, was purified by distillation from CaH_2_. Melting and decomposition points were measured with a hot-stage apparatus with a melting point measuring device MP-J3 (Yanako, Kyoto, Japan) and were uncorrected. Elemental analyses were performed at the Graduate School of Science, Osaka University (Toyonaka, Japan). ^1^H NMR spectrum was obtained on an ECA-500 spectrometer (JEOL, Akishima, Japan) with DMSO-*d*_6_ using Me_4_Si as an internal standard. Infrared spectrum was recorded on an FT/IR-660 Plus spectrometer (JASCO, Hachioji, Japan) using a KBr plate (resolution 4 cm^−1^). UV-Vis absorption spectra were measured on a UV-Vis scanning spectrophotometer UV-3100PC (Shimadzu, Kyoto, Japan). High-resolution mass spectra (FAB-MS) were measured on a double-focusing magnetic sector mass spectrometer JMS-700 (JEOL, Akishima, Japan). XRD measurement was performed on a SmartLab X-ray diffractometer (Rigaku, Akishima, Japan) using Cu Kα radiation at 45 kV and 200 mA (*λ* = 1.5415 Å) at room temperature.

### 4.2. Synthesis

#### 4.2.1. Synthesis of Tetra-*n*-hexylammonium 2,6,10-tri-4′-pyridyl-4,8-dioxo-4H,8H-dibenzo[*cd,mn*] pyren-12-olate [(*n*-Hex)_4_N^+^)(**4^−^**)]

To a three-necked flask, **3a** (500 mg, 0.89 mmol), 4-(4′,4′,5′,5′-tetramethyl-1′,3′,2′-dioxaborolan-2′-yl)pyridine (1.83 g, 8.94 mmol), Cs_2_CO_3_ (1.75 g, 5.37 mmol) were added under argon atmosphere, and then dissolved in distilled DMF (150 mL). Pd(PPh_3_)_4_ (105 mg, 0.09 mmol) was added to the mixture, and stirred at 100 °C under argon for 10 h. To the reaction mixture, 2 M HCl aq (~3 mL) was added, and the resulting precipitate was filtrated, and washed with methanol and dried at 60 °C for 5 h. The resulting product (589 mg) still contained a few percent of impurities according to ^1^H NMR. The crude product was suspended into methanol and tetra-*n*-hexylammonium hydroxide (~10% in methanol solution) was added, and then stirred for 3 h. The reaction mixture was changed from purple suspension to green solution. The mixture was concentrated under reduced pressure, and the residue was washed with ethyl acetate to give [(*n*-Hex)_4_N^+^)(**4^−^**)] (629 mg, 77%) as a green solid. Recrystallization from DME/hexane resulted in a fine crystal suitable for single crystal X-ray diffraction analysis. melting point (mp). 154–156 °C; ^1^H NMR (500 MHz, DMSO-*d*_6_): *δ* 0.86 (t, *J* = 6.5 Hz, 12H), 1.27 (t, *J* = 6.5 Hz, 24H), 1.54 (m, 8H), 3.13 (m, 8H), 7.82 (dd, *J* = 4.5 Hz, 1.5 Hz, 6H), 8.67 (dd, *J* = 4.5 Hz, 2.0 Hz, 6H), 8.90 (s, 6H) ppm; UV-Vis (CH_2_Cl_2_): *λ*_max_ (ε/M^−1^ cm^−1^) 333 (19,300), 440 (43,500), 602 (10,500), 624 (11,400), 664 (16,100), 681 (15,300) nm; IR (KBr): *ν*1242, 1292, 1486, 1598, 1614, 2859, 2953 cm^−1^; FAB-MS (negative mode) (%intensity): C_37_H_18_N_3_O_3_: Calcd: 552.1348. Found: 552.1354 (100%); Anal. Calcd for (C_61_H_70_N_4_O_3_)(H_2_O)_2_: C, 77.67; H, 7.91; N, 5.92%. Found: C, 77.49; H, 7.72; N. 5.82%.

#### 4.2.2. Synthesis of Lithium 2,6,10-tri-4′-pyridyl-4,8-dioxo-4H,8H-dibenzo[*cd,mn*]pyren-12-olate [Li^+^**4^−^**]

[(*n*-Hex)_4_N^+^](**4^−^**) (224 mg, 0.247 mmol) was placed in a round bottled flask and dissolved with methanol (60 mL). To this solution, 2 M HCl aq (60 mL) was added at room temperature and stirred for 3 h. The green solution was changed to a purple suspension. The precipitate was collected by filtration and washed with water and methanol to give a brown solid (103 mg). The resulting solid was suspended in methanol in round bottled flask and LiOH•H_2_O (200 mg, 4.55 mmol) was added to the mixture and stirred for 3 h under Ar. The mixture changed from a purple suspension to a green solution. The reaction mixture was evaporated under reduced pressure to dryness. The residue was washed with water and collected by filtration. After washing with a small amount of methanol, the product was dried under reduced pressure at 50 °C for 2 h. (81.2 mg, 78%). ^1^H NMR (500 MHz, DMSO-*d*_6_): *δ* 7.93 (dd, *J* = 6.0 Hz, 3.0 Hz, 6H), 8.71 (dd, *J* = 5.5 Hz, 2.0 Hz, 6H), 9.10 (s, 6H) ppm; UV (KBr): *λ*_max_ 268, 429, 632 nm; IR (KBr): *ν*1247, 1295, 1487, 1542, 1561, 1600 cm^−1^; Anal. Calcd for (LiC_37_H_18_N_3_O_3_)(H_2_O)_6_: C, 66.59; H, 4.53; N, 6.29%. Found: C, 66.54; H, 4.59; N, 6.16%.

#### 4.2.3. Synthesis of 2,6,10-tri-4′-pyridyl-4,8-dioxo-4H,8H-dibenzo[*cd,mn*]pyren-12-oxyl [**4**]

In a 200-mL round bottomed flask, DDQ (46.7 mg, 0.21 mmol) was dissolved in CH_2_Cl_2_ (20 mL). To this mixture, a solution of [(*n*-Hex)_4_N^+^](**4^−^**) (20.2 mg, 0.022 mmol) in CH_2_Cl_2_ (10 mL) was added slowly. The mixture was stirred at room temperature for 2 h. The resulting precipitate was collected by filtration, and then washed with CH_2_Cl_2_, to give the neutral radical **4** (12.1 mg, 98%) as a black powder. decomposition point (dp). > 300 K (in air); UV (KBr): *λ*_max_ 259, 527 nm; IR (KBr): *ν*1222, 1311, 1482, 1612, 1712 cm^−1^.

#### 4.2.4. Synthesis of [Zn^2+^(4^−^)(DMSO)_2_](NO_3_^−^)(solvent)_x_ [zinc(II) complex **5**]

The single crystals of **5** were grown from a double-layered solution consisting of an ethanol solution of Zn(NO_3_)•6H_2_O (5.0 mM) as the top layer, and a DMSO/nitrobenzene solution (1:1) of Li^+^**4^−^** (1.0 mM) as the bottom layer in *ϕ* 8 mm × 20 mm length glass tubes. After slow diffusion of the solution at room temperature for 2 weeks, green crystals suitable for X-ray crystal structure analysis appeared. During the diffusion, not only crystals of complex **5** but also a small amount of insoluble powder, which may be the polymorphs, appeared. This fine powder could be removed by dispersing the crude product in a solvent and the decantation, and the pure crystals were obtained by filtration followed by the drying in vacuo. UV (KBr): *λ*_max_ 270, 447, 620(sh) nm; IR (KBr): *ν*1248, 1296, 1385, 1487, 1561, 1601 cm^−1^; Anal. Calcd for C_41_H_30_N_4_O_8_S_2_Zn: C, 58.89; H, 3.62; N, 6.70%. Found: C, 59.18; H, 4.09; N, 6.25%.

### 4.3. X-Ray Crystallography

X-Ray single crystal diffraction analyses were performed on a Rigaku-Raxis imaging plate system (Rigaku, Akishima, Japan) or a BRUKER-APEX X-Ray diffractometer (BRUKER-Japan, Osaka, Japan) equipped with a CMOS (Complementary Metal Oxide Semiconductor) detector with graphite monochromated Mo Kα or Cu Kα, respectively. The structure was determined by a direct method using the SHELXS program [65], and refinement was performed by a full-matrix least-squares on *F*^2^ using SHELXL-2014 [66]. All atoms except hydrogen atoms were refined using anisotropic thermal factors, and all hydrogen atoms were included in the calculation without refinement. We applied the empirical absorption correction. CCDC-2005846 and 2005847 contains the supplementary crystallographic data for this paper. These data can be obtained free of charge from The Cambridge Crystallographic Data Centre.

#### 4.3.1. Crystal Data for [(n-Hex)_4_N^+^)](**4^−^**)(H_2_O)_2.5_(DME)_0.5_

C_252_H_320_N_16_O_26_, *M*_r_ = 3989.22, monoclinic, *P*2_1_/*c* (#14), *a* = 21.8447(18) Å, *b* = 22.339(2) Å, *c* = 22.729(2) Å, *β* = 102.829(2)°, *V* = 10814.3(17) Å^3^, *T* = 130 K, *Z* = 2, *d*_calc_ = 1.225 g cm^−3^, *µ* = 0.078 mm^−1^, 2*θ*_max_ = 135.28°, *λ*(MoKα) = 0.71075 Å, ω scan mode, 81,875 reflections, of which 24,595 were unique and 6586 were included in the refinement [*I* > 2.00 σ(*I*)], data corrected for Lorentzian and polarization effects; an empirical absorption correction resulted in transmission factors ranging from 0.925 to 0.984. The final values were *R*_1_ = 0.1045, _w_*R*_2_ = 0.3554, and *GOF* (*Goodness-of-Fit*) = 0.912, and the maximum positive and negative peaks in the *∆F* map were *ρ*_max_ = 0.564 e Å^−3^ and *ρ*_min_ = −0.312 e Å^−3^. The disorder of the DME molecules and flexible *n*-hexyl groups caused the large w*R*_2_ value.

#### 4.3.2. Crystal Data for [Zn^2+^(**4^−^**)(DMSO)_2_](NO_3_^−^)(solvent)_x_ **5**

C_41_H_24_N_3_O_14_S_2_Zn, *M*_r_ = 912.14, monoclinic, *C*2/*c* (#15), *a* = 15.0694(4) Å, *b* = 31.7869(9) Å, *c* = 9.9300(3) Å, *β* = 118.824(2)°, *V* = 4167.2(2) Å^3^, *T* = 93 K, *Z* = 4, *d*_calc_ = 1.454 g cm^−3^, *µ* = 2.371 mm^−1^, 2*θ*_max_ = 136.68°, *λ*(CuKα) = 1.54178 Å, *ω* scan mode, 21,303 reflections, of which 3746 were unique and 3237 were included in the refinement [*I* > 2.00 *σ*(*I*)], data corrected for Lorentzian and polarization effects; an empirical absorption correction resulted in transmission factors ranging from 0.9198 to 0.9944. The final values were *R*_1_ = 0.0990, _w_*R*_2_ = 0.2886 and *GOF* = 1.046, and the maximum positive and negative peaks in the *∆F* map were *ρ*_max_ = 2.647 e Å^−3^ and *ρ*_min_ = −1.385 e Å^−3^. Due to the severe disorder of NO_3_^−^ and solvent molecules within the cavity, these atoms could not be determined, and large residual densities remained in the refinement.

### 4.4. Electrochemical Measurements

Electrochemical measurements in the solution state were conducted on an ALS electrochemical analyzer model 630A (BAS, Tokyo, Japan). An Ag/AgNO_3_ electrode in acetonitrile served as reference electrode, a 3 mm diameter glassy carbon electrode as counter electrode, and a Pt wire as working electrode. CV and DPV measurements were performed at room temperature under Ar in dry DMF containing 0.1 M (*n*-Bu)_4_N^+^ClO_4_^−^ as a supporting electrolyte. The redox potentials were finally calibrated against the ferrocene/ferrocenium couple.

### 4.5. Electrochemical Absorption Spectroscopy

UV-Vis/near-IR absorption spectra during electrochemical oxidation were measured at room temperature using an ultra-high resolution spectrometer HR4000 (Ocean Optics, Tokyo, Japan) with a DH-2000-BAL UV-Vis-NIR light source (Ocean Optics, Tokyo, Japan), a 1 mm wide cell equipped with fine-mesh platinum as a working electrode, Pt wire as a counter electrode, and an Ag/AgNO_3_ as a reference electrode at suitable external potentials that could generate the oxidized species in CH_2_Cl_2_ in the presence of 0.1 M (*n*-Bu)_4_N^+^PF_6_^−^.

### 4.6. Battery Fabrication, Solid-State CV Measurement, and Charge–Discharge Experiments

Cathode composites of coin-type cells containing 10 wt% active cathode material were fabricated by mixing 10:80 (wt%) of zinc(II) complex **5** and AB. The materials were thoroughly ground in a mortar and dispersed in *N*-methyl-2-pyrrolidone (NMP) containing PVDF (10 wt%), and then kneaded with an ARE-310 centrifugal mixer (Thinky, Tokyo, Japan). A cathode sheet was prepared by pressing the composite using a PI-1201 filmcoater (TA Instruments, Tokyo, Japan) on an Al sheet, followed by drying in vacuo at 120 °C for 1 h. The sheet was cut out to a diameter of 12 mm and used as a cathode disk. In a coin-type cell, a Li metal plate, a porous polymer film separator and the cathode disk were stacked, and the resulting cell was filled with the electrolyte solution in an argon-filled glove box. The electrolyte solution was a 1 M LiPF_6_ solution in a mixture of ethylene carbonate (EC) and diethyl carbonate (DEC) in a 3:7 ratio (vol/vol%). A VMP3 multichannel potentiostat (Bio-Logic Science Instruments, Grenoble, France) was used for the solid-state CV and galvanostatic discharge–charge experiment.

## 5. Conclusions

Tripyridyl-**TOT 4** as a tridentate ligand with three-fold symmetry successfully formed a new redox-active MOF with a zinc(II) ion. Complex **5** formed a 2D coordination network with a cavity and exhibited a multistage redox ability in the solid state. Although the ORB using **5** as a cathode active material had a small capacity due to the collapse of the crystal structure, the robust coordination bond of **4** demonstrated the improvement of the cycle performance. Although the 2D coordination network and cavity of complex **5** did not show sufficient strength, these experimental results indicate that the combination of **TOT** and a porous structure will highly contribute to the development of various electronic materials such as energy storage by providing an intriguing strategy for the material design. The most striking feature of **TOT** is the extremely high stability of the neutral radical species and its strong intermolecular singly occupied MO (SOMO)–SOMO interaction [41]. As shown in complex **5**, by controlling the arrangement of the **TOT** skeletons by coordination bonds, new electrical and chemical functions can be explored, where the magnetic and/or electrical properties are deeply linked with inclusion behavior of the cavity. Studies using a redox-active porous coordination polymer as an electrochemical catalyst for oxygen reduction and oxygen evolution reactions, for example, have recently attracted much attention [25,67]. In addition, the recent theoretical study by Bredas and co-workers suggested that 2D π-conjugated covalent organic frameworks composed of **TOT** possess a high potential to exhibit intriguing electrical/magnetic properties [68]. We are currently continuing to research these new unconventional materials, including an application for a molecular catalyst [46].

## Supplementary Materials

The following are available online at https://www.mdpi.com/1422-0067/21/13/4723/s1, Figure S1: ^1^H NMR spectrum of [(***n*-Hex**)**_4_N^+^**](**4^−^**) in DMSO-*d*_6_, Table S1: Intramolecular C–C and C–O bond lengths (Å) and dihedral angles (°) between **TOT** and pyridyl moieties of **4^−^** in the crystal structures, Figure S2: Cyclic voltammograms of [(***n*-Bu**)**_4_N^+^**(**1^−^**) and [(***n*-Hex**)**_4_N^+^**](**4^−^**) in DMF, Figure S3: Asymmetric unit in the crystal structure of [(*n*-Hex)_4_N^+^)](**4^−^**)(H_2_O)_2.5_(DME)_0.5_, Figure S4: Molecular structure in [Zn^2+^(**4^−^**)(DMSO)_2_](NO_3_^−^)(solvent)_x_ **5** with the atomic numbering scheme, Figure S5: XRD spectrum of the complex **5**.
