# Supplementary material for: 2D Coordination Network of Trioxotriangulene with Multiple Redox Abilities and Its Rechargeable Battery Performance
Source: Int J Mol Sci. 2020 Jul 2;21(13):4723. doi: 10.3390/ijms21134723 (PMC7369800; doi:10.3390/ijms21134723)
Supplement: Supplementary file 1 [file ijms-21-04723-s001.pdf]

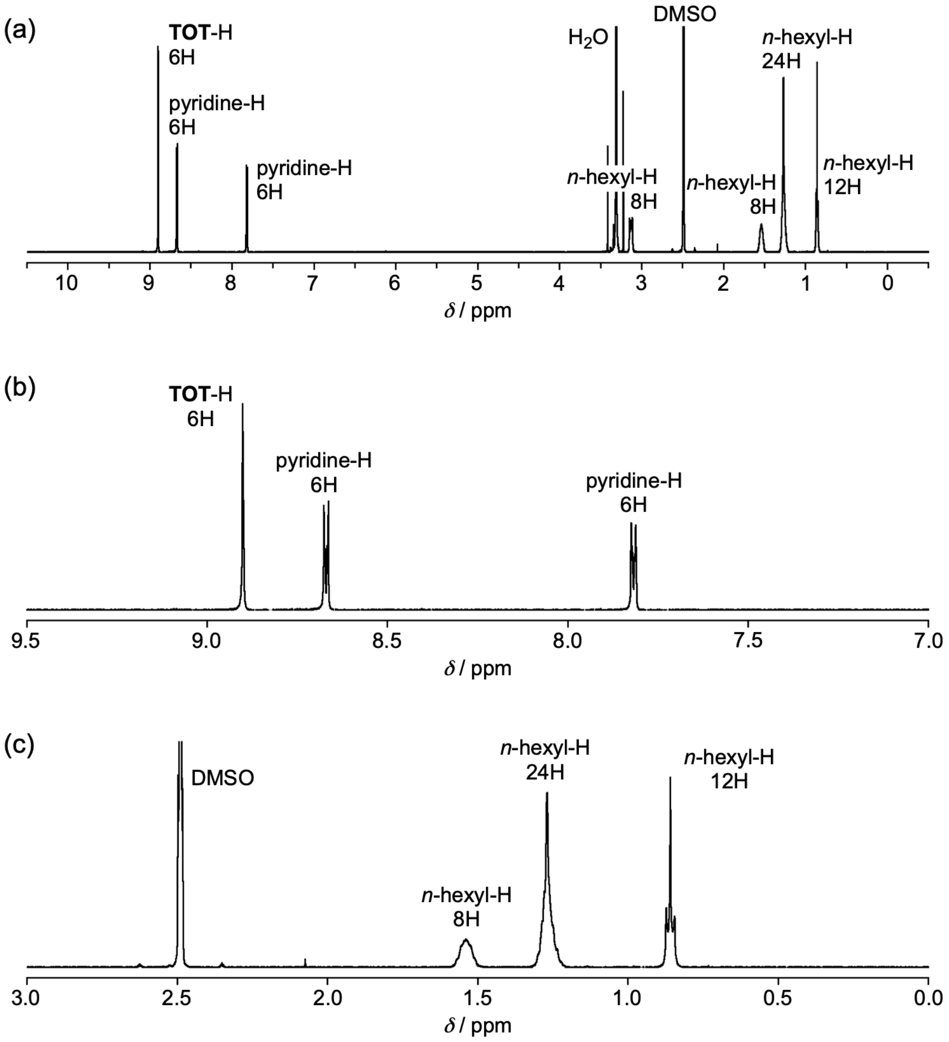

Figure S1. ^{1}H NMR spectrum of **[(*n*-Hex)$_4$N$^+$](4$^-$)** in DMSO-d_6. (a) Full measurement area (δ = –0.5–10.5 ppm), (b) Low magnetic field region (δ = 7.0–9.5 ppm). (c) High magnetic field region (δ = 0.0–3.0 ppm).

Table S1. Intramolecular C–C and C–O bond lengths (Å) and dihedral angles (°) between **TOT** and pyridyl moieties of **4**$^-$ in the crystal structures.

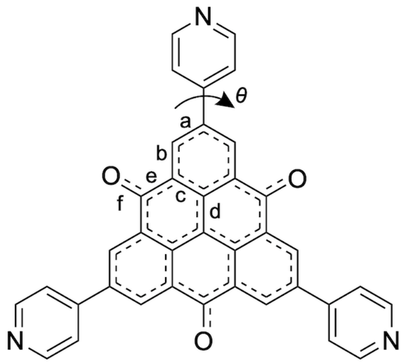

	**[(*n*-Hex)$_4$N$^+$](4$^-$)-A***	**[(*n*-Hex)$_4$N$^+$](4$^-$)-B***	**[Zn^{2+}(4$^-$)(DMSO)$_2$](NO$_3^-$)**
a/Å	1.404(7), 1.380(7)	1.407(7), 1.400(7)	1.397(6), 1.403(8)
	1.386(8), 1.380(7)	1.384(8), 1.399(7)	1.393(8)
	1.385(7), 1.394(7)	1.395(7), 1.367(7)	
b/Å	1.382(7), 1.388(7)	1.378(6), 1.382(6)	1.375(7), 1.395(7)
	1.388(7), 1.413(7)	1.369(7), 1.382(7)	1.396(8)
	1.368(7), 1.359(7)	1.399(7), 1.389(7)	
c/Å	1.430(8), 1.415(6)	1.436(7), 1.413(7)	1.427(6), 1.420(7)
	1.417(8), 1.424(7)	1.417(7), 1.430(6)	1.422(7)
	1.443(7), 1.421(7)	1.425(7), 1.421(6)	
d/Å	1.419(7), 1.414(7)	1.407(6), 1.416(7)	1.416(9), 1.420(6)
	1.404(7)	1.415(7)	
e/Å	1.456(7), 1.460(7)	1.479(7), 1.481(7)	1.473(7), 1.471(7)
	1.480(8), 1.455(7)	1.461(7), 1.453(7)	1.467(7)
	1.445(8), 1.463(7)	1.459(7), 1.469(7)	
f/Å	1.250(5), 1.249(6)	1.258(6), 1.227(7)	1.240(6), 1.234(9)
	1.241(6)	1.257(5)	
θ/°	32.6, 23.3, 23.6	31.1, 23.5, 24.3	37.8, 29.3

* Two crystallographically independent molecules (**A** and **B**) were found in the crystal structure.

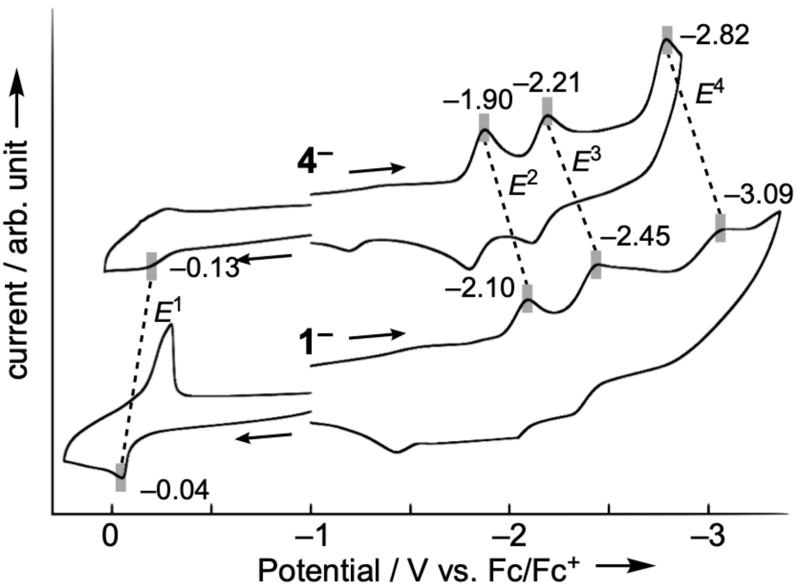

Figure S2. Cyclic voltammograms of **[(*n*-Bu)$_4$N$^+$](1$^-$)** and **[(*n*-Hex)$_4$N$^+$](4$^-$)** in DMF (5 and 3 mM, respectively). Due to the poor solubility of polyanionic and neutral radical species, the redox processes E^4 and E^1 were irreversible, and peak potentials are listed. The results were calibrated with Fc/Fc$^+$ couple.

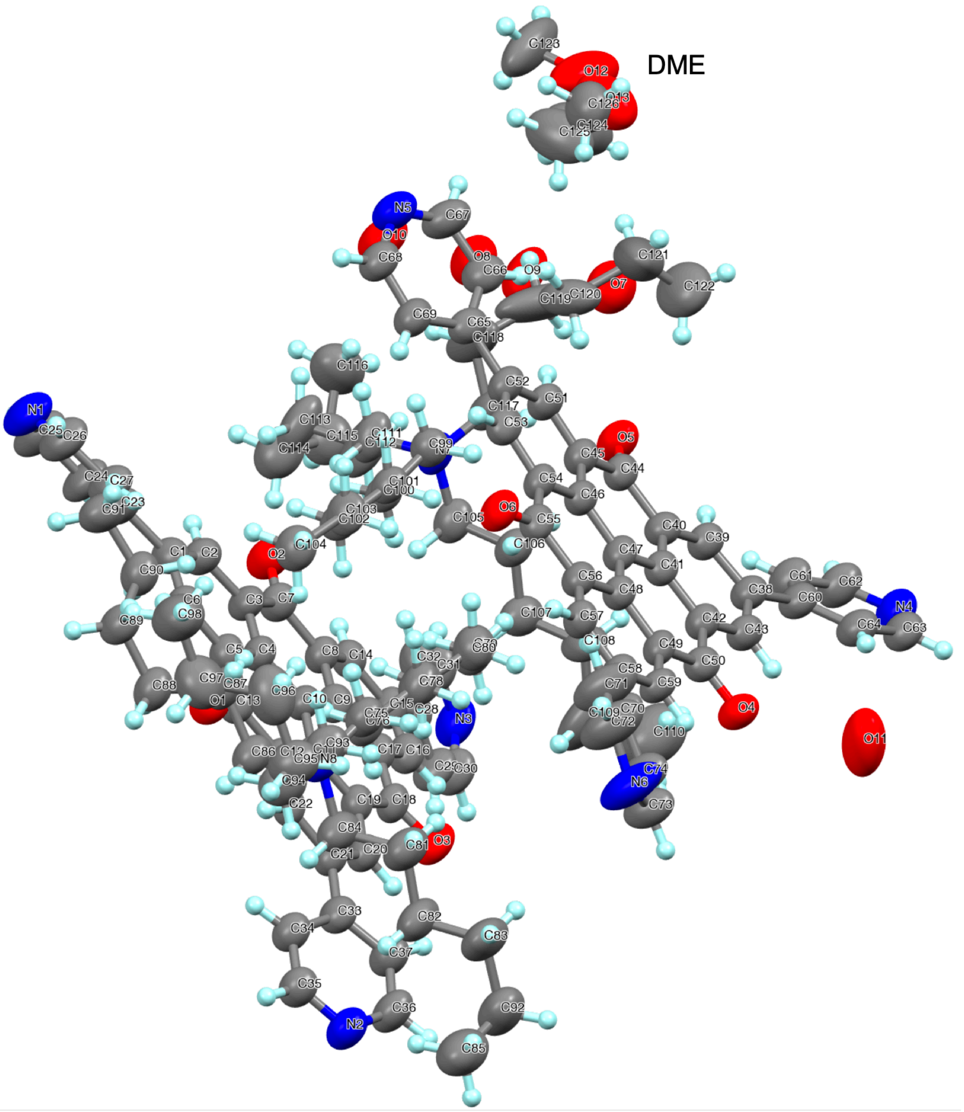

Figure S3. Asymmetric unit in the crystal structure of $[(n\text{-Hex})_4N^+)](\mathbf{4}^-)(H_2O)_{2.5}(DME)_{0.5}$ with the atomic numbering scheme. Color code: carbon (gray), nitrogen (blue), oxygen (red).

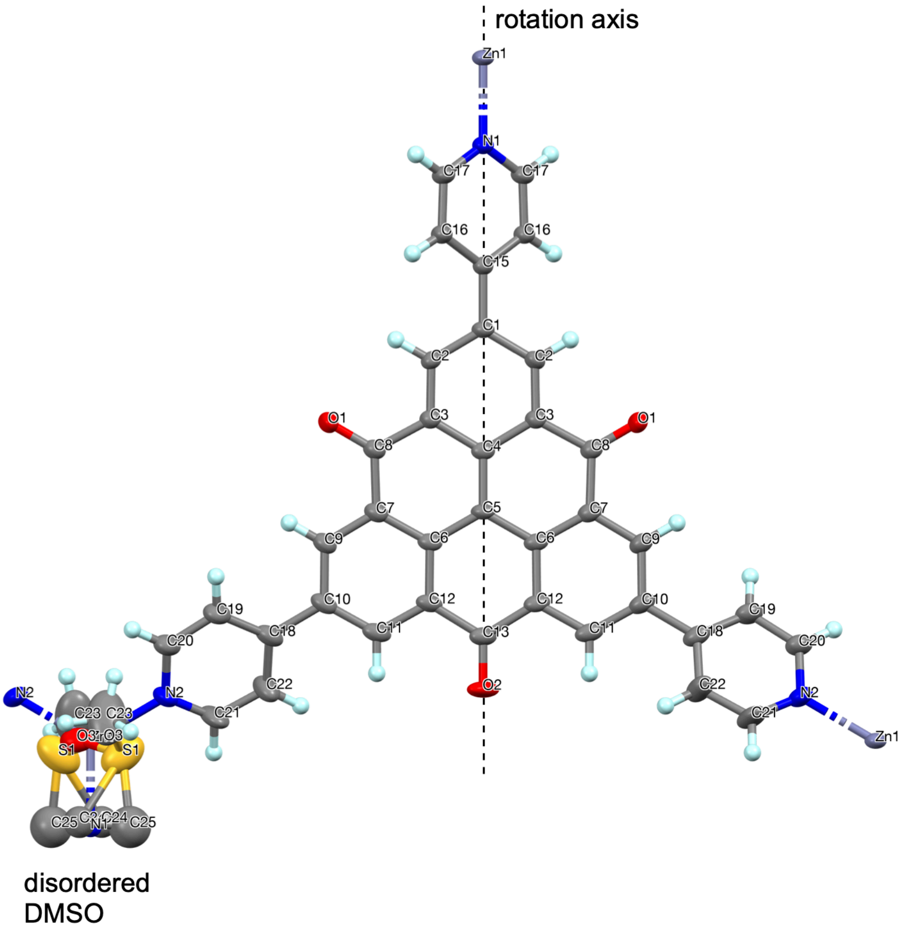

Figure S4. Molecular structure in $[Zn^{2+}(\mathbf{4}^{-})(DMSO)_2](NO_3^{-})(solvent)_x$ **5** with the atomic numbering scheme. Color code: carbon (gray), nitrogen (blue), oxygen (red), sulfur (yellow), zinc (blue gray). The half of the molecule is asymmetric.

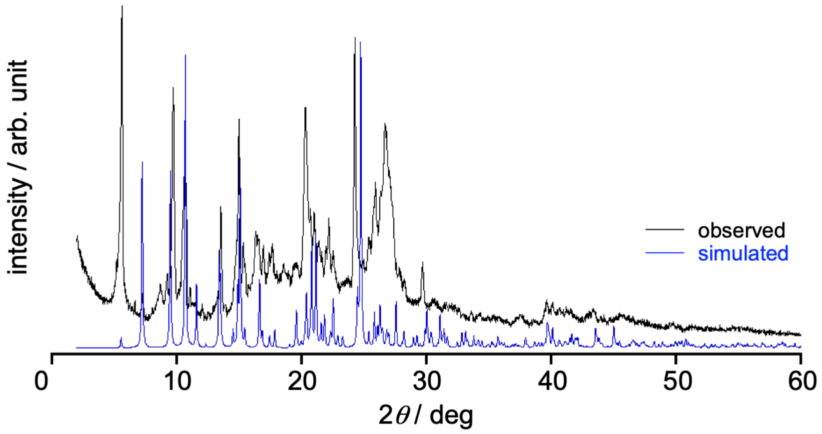

Figure S5. XRD spectrum of the complex **5**. Black line: observed spectra, blue lines: simulated spectrum from the structure of single crystal X-ray analysis. The sample of **5** was dried in vacuo at 60 °C before measurement.
